# Profiles of cytokines secreted by isolated human endometrial cells under the influence of chorionic gonadotropin during the window of embryo implantation

**DOI:** 10.1186/1477-7827-11-116

**Published:** 2013-12-17

**Authors:** Akhilesh Srivastava, Jayasree Sengupta, Alka Kriplani, Kallol K Roy, Debabrata Ghosh

**Affiliations:** 1 Department of Physiology, All India Institute of Medical Sciences, New Delhi, India; 2 Department of Obstetrics and Gynaecology, All India Institute of Medical Sciences, New Delhi, India; 3 Present address: Department of Physiology, North DMC Medical College, Hindu Rao Hospital, New Delhi 110007, India

**Keywords:** Cell culture, Cytokines, Endometrium, Epithelial cells, hCG, Immunoblot, Multiplexing, RT-PCR, Stromal cells

## Abstract

**Background:**

Several studies have indicated that human pre-implantation embryo-derived chorionic gonadotropin (hCG) may influence the implantation process by its action on human endometrial epithelial and stromal cells. Despite reports indicating that hCG acts on these cells to affect the production of several cytokines and growth factors (e.g., MIF, IGF-I, VEGF, LIF, IL-11, GMCSF, CXL10 and FGF2), our understanding of the integral influence of hCG on paracrine interactions between endometrial stromal and epithelial cells during implantation is very limited.

**Methods:**

In the present study, we examined the profile of 48 cytokines in the conditioned media of primary cell cultures of human implantation stage endometrium. Endometrial epithelial cells (group 1; n = 20), stromal cells (group 2; n = 20), and epithelial plus stromal cells (group 3; n = 20) obtained from mid-secretory stage endometrial samples (*n* = 60) were grown on collagen and exposed to different doses (0, 1, 10 and 100 IU/ml) of rhCG for 24 h *in vitro.* Immunochemical and qRT-PCR methods were used to determine cytokine profiles. Enrichment and process networks analyses were implemented using a list of cytokines showing differential secretion in response to hCG.

**Results:**

Under basal conditions, endometrial epithelial and stromal cells exhibited cell type-specific profiles of secreted cytokines. Administration of hCG (100 IU) resulted in significantly (P < 0.05) different cytokine secretion profiles indicative of macropinocytic transport (HGF, MCSF) in epithelial cells, signal transduction (CCL4, FGF2, IL-1b, IL-6, IL-17, VEGF) in stromal cells, and epithelial-mesenchymal transition (FGF2, HGF, IL-1b, TNF) in mixed cells. Overall, the administration of hCG affected cytokines involved in the immune response, chemotaxis, inflammatory changes, proliferation, cell adhesion and apoptosis.

**Conclusions:**

CG can influence the function of the endometrium during blastocyst implantation via its differential action on endometrial epithelial and stromal cells. CG may also affect complex paracrine processes in the different endometrial cell types.

## Background

Blastocyst implantation in humans involves a complex process that occurs during the mid-luteal phase of the menstrual cycle when the maternal endometrium becomes receptive and the blastocyst becomes free of the zona pellucida and invades the endometrium. There is substantial evidence to suggest that mutual interaction between pre-implantation stage endometrium and the embryo plays a critical role in this process [1]. One of the factors that may potentially influence the biology of the peri-implantation stage endometrium is human chorionic gonadotropin (hCG) [1], which may be secreted by the human pre-implantation embryo [2,3] and implantation-stage human endometrium [4,5]. This secretion profile of hCG is supported by the observation that in the secretory phase, epithelial and stromal cells from the human endometrium express receptors for hCG [6,7]. Furthermore, it has been demonstrated that the administration of hCG can influence endometrial functions in various experimental models [7-13].

Our understanding of the integral influence of hCG in paracrine interactions between endometrial stromal and epithelial cells during implantation is limited. In a recent study, Paiva et al. [14] examined the effect of hCG (0.2, 2 and 20 IU/ml) administered to human endometrial epithelial cells *in vitro* and observed that recombinant hCG stimulated the secretion of six analytes (VEGF, LIF, IL-11, GMCSF, CXL10 and FGF2). However, hCG acts on human endometrial stromal cells to promote a variety of functions that include stimulation of production of the multi-functional cytokine, macrophage inhibitory factor (MIF) [9], suppression of the cellular apoptotic machinery [15,16], and reduction in insulin-like growth factor-I (IGF-I) and interferon-gamma-mediated responsiveness of stromal decidual cells [11,17]. Nonetheless, the responsiveness of human endometrial stromal cells and epithelial cells to hCG remains undefined mainly for two reasons. First, the endometrial cells used in previous studies were grown on a conventional two-dimensional plastic substratum, which typically fails to support a physiological phenotype. Several studies have indicated that a three-dimensional culture system is a better model in this regard [18-20]. Also, the integral influence of paracrine interactions between stromal and epithelial cells in mediating hCG effects has not been reported [20]. In the present study, we addressed these issues through the multi-analyte profiling of 48 cytokines, chemokines and growth factors. The secretion of these factors was assessed in the conditioned media of three-dimensional primary cell cultures of human endometrial epithelial cells, stromal cells, and epithelial plus stromal cells isolated from endometrial biopsies collected during the ‘window’ of implantation. These cell types were grown on collagen-I biomatrix and exposed to different doses of hCG. The cytokines, chemokines and growth factors investigated in the study have been previously reported to be synthesised and secreted by the human endometrium [21].

## Methods

### Patients and tissue collection

This study was approved by the Ethics Committee of the All India Institute of Medical Sciences (AIIMS) and conducted in accordance with the Helsinki Declaration. Sixty pre-menopausal women (mean age: 28 ± 4 years; BMI: 18.9-22.8 kg/m^2^) with regular menstrual cycles (30 ± 2 days) attending the out-patient fertility clinic of the Department of Obstetrics and Gynaecology, AIIMS, were selected for the present study. The women underwent dilation and curettage to collect endometrial tissue samples for diagnostic gynaecological procedures and were *a priori* screened to determine whether they were negative for pregnancy and tuberculosis. Also, these women had not received any steroid treatment for at least 4 months prior to tissue collection and were not suffering from any endocrine disorder or systemic disease. Randomly chosen pieces of sample were provided to us, and all women provided written informed consents to participate in the study. The tissue samples were collected on ice in sterile DMEM:F12 (1:1) medium containing 5% (v/v) FCS, gentamicin (10 μg/ml), penicillin (100 IU/ml), streptomycin (100 μg/ml) and fungizone (2.5 μg/ml) and were immediately transported on ice to the cell culture laboratory for further processing.

Each fresh specimen was washed three times in ice-cold sterile HBSS supplemented with antibiotics and an antimycotic agent to remove blood and secretions and were randomly divided into two parts. One small section was washed in phosphate buffer saline (pH 7.4) and fixed in ice-cold neutral buffered paraformaldehyde (4% w/v), pH 7.4, for histological assessment, and the residual tissue was processed for tissue dissociation and cell separation as described below.

### Cell isolation and separation

Enzymatic dissociation of endometrial tissue followed by cell separation was performed using previously described standardised procedures [19,22,23]. Briefly, the endometrial specimen was washed in HBSS, minced and incubated at 37°C with collagenase III (0.2%, w/v) in HBSS supplemented with 2% (v/v) foetal calf serum (FCS), 15 mM HEPES (N-2-hydroxyethyl-piperazine-N’-2-ethanesulfonic acid), 1X antibiotic-antimycotic solution [penicillin (100 IU/ml), streptomycin (100 μg/ml) and fungizone (2.5 μg/ml)] and gentamicin (10 μg/ml) for 30 minutes with shaking at 50 rpm. The final cell suspension contained primarily single stromal cells and fragments of glands. The cell suspension was washed three times with Ca^++^/Mg^++^-free HBSS supplemented with the antibiotic/antimycotic mixture (HBSS-mod) and filtered through a sterile, pre-equilibrated mesh filter (51 μm). The filtrate contained the ‘stromal cell enriched’ fraction. The residual ‘epithelial cell enriched’ fraction was further purified by unit gravity sedimentation in 10% (v/v) FBS. The stromal cells remained in cell suspension, and the sediment, consisting of sheets, fragments and clumps of epithelial cells, was collected and washed three times with HBSS-mod. The ‘epithelial cell fraction’ was further enriched by density-dependent fractionation on a discontinuous Percoll gradient as described previously [19,22,23]. The ‘epithelial cell enriched fraction’ was re-suspended in complete medium (DMEM:F12 with 10% FCS) and stored on ice.

The ‘stromal cell enriched fraction’ was further enriched by density-dependent fractionation on a discontinuous Percoll gradient as described previously [19,22,23]. CD45-positive leucocytes were depleted using anti-CD45 MACS microbeads and LS magnetic separation columns in a MidiMACS separator (Miltenyi Biotec, Bergisch Gladbach, Germany) as described [24]. The negative fraction containing the stromal cell-enriched population was washed twice with HBSS-mod at 300 × g for 10 minutes and re-suspended in 1 ml of complete medium (DMEM:F12 with 10% FCS) and kept on ice. For separated mixed cells, endometrial cells separated by enzymatic digestion were not passed through a mesh filter, but the remaining steps were followed as described above. The yield and viability of the isolated cells were determined using standard protocols [24]. The relative abundance of cytokeratin-positive, vimentin-positive, CD45-positive and vW factor-positive cells in all three groups was determined using standard immunocytochemical procedures [25].

### Primary culture of human endometrial cells

Primary cell cultures at 1x10^5^ cells/cm^2^ were separated into three different groups: Group 1: epithelial cells (n = 20), Group 2: stromal cells (n = 20), and Group 3: mixed cells (n = 20). All three groups were cultured in DMEM:F12 (1:1) medium containing 10% (v/v) FCS, gentamicin (10 μg/ml), penicillin (100 IU/ml), streptomycin (100 μg/ml) and fungizone (2.5 μg/ml) in a 5% CO_2_ atmosphere at 37°C on rat-tail collagen type I as described previously [19,22,23]. Briefly, rat-tail collagen was extracted from tendon bundles of sterilised rat tails in sterile diluted (1:1,000) acetic acid at 4°C, and the acid-soluble collagen (2.0 ± 0.2 mg/ml) was collected aseptically and used as stock as previously described [19,22]. Collagen biomatrix was generated by neutralising acid collagen with 0.34 N NaOH and diluted with 10X medium to a concentration of 1.2 ± 0.1 mg/ml at pH 7.4 on ice and then gelled at 37°C with 5% carbon dioxide [19,22]. Cells were allowed to attach with a daily change of medium; at 72 hours when cells had grown to ≈ 80% confluence, serum-free DMEM:F12 (1:1) medium supplemented with insulin (10 μg/ml), transferrin (5.5 μg/ml), selenium (6.7 ng/ml), hydrocortisone (5 μg/ml), gentamicin (10 μg/ml), penicillin (100 IU/ml), streptomycin (100 μg/ml) and fungizone (2.5 μg/ml) (designated as *basal condition*) was added. Serum-free cell cultures in all three groups were exposed to different concentrations (0, 1, 10, 100 IU/ml) of rhCG in triplicate. After 24 hours, cultures were terminated, and gels containing cells were used for RNA extraction and quantitative gene expression using RT-PCR. The remaining conditioned media were collected and centrifuged at 10,000 × g for 5 minutes, and the supernatant was stored at -80°C for immunoassay of multiple cytokines and immunoblotting experiments. Five replicates in duplicate were conducted for each set of experiments.

### hCG binding to isolated endometrial cells

Different sets (n = 3) of experiments were performed to examine the binding of biotinylated rhCG to the cells grown in culture. Biotin labelling of hCG was performed using a commercially available biotin labelling kit from Thermo Scientific (Rockford, IL, USA) according to the manufacturer’s protocol. Isolated endometrial cells were grown separately on collagen-coated cover slips in DMEM:F12 complete medium as described above. The cells were then washed twice in serum-free DMEM:F12 and exposed to biotinylated rhCG (200 mIU/ml) at 37°C in a humidified incubator with 5% CO_2_ for 40 minutes; these parameters were determined based on initial optimisation with three concentrations (100, 200 and 400 mIU/ml) of biotinylated rhCG and two time points (20 minutes and 40 minutes). To assess the specificity of hCG binding, parallel cultures of attached cells after 72 hours of culture were incubated with biotinylated scrambled sham rhCG, unconjugated biotin, unconjugated rhCG in place of biotinylated rhCG or without any of these reagents. The incubation was stopped with ice-cold PBS, and the cells were fixed in 4% (w/v) paraformaldehyde to assess the bound hCG by immunofluorescence using an Alexa488-conjugated anti-biotin monoclonal antibody and a Confocal Laser Scanning Microscope (DMIRE2, Leica Microsystems, Wetzlar GmbH, Germany) [24,25].

### Multiplex assays of cytokines in conditioned media

The concentrations of 48 cytokines, chemokines and growth factors (for details, see Additional file 1: Table S1) that have been reported to be synthesised and secreted by the human endometrium [21] were analysed. Cell culture supernatants were assessed by quantitative cytokine assays using a Bioplex^TM^ Pro-human cytokine 27-plex panel and a cytokine 21-plex panel based on xMAP technology (Bio-Rad Laboratory, Hercules, CA, USA) according to the pre-optimised protocol. Briefly, antibody–conjugated beads were added to individual wells of a 96-well filter plate and adhered using vacuum filtration. After washing, 50 μl of prediluted standards or serum were added, and the filter plate was shaken at 300 rpm for 30 minutes at room temperature. A prediluted multiplex biotin-conjugated detection antibody was then added for 30 minutes. Prediluted streptavidin-conjugated PE was added followed by an additional wash and the addition of Bio-Plex assay buffer. The filter plate was analysed, and concentrations of each cytokine were determined using the BioRad BioPlex 200 instrument equipped with BioManager v6.0 software (Bio-Rad). All samples were run in duplicate. Standard curves were generated for each biomarker. Goodness of fit for standard curves was determined by the standard recovery method and by calculating the concentration of each standard [26].

### Western blotting

Based on the post-hoc analysis of the immunoassays as described below, 13 proteins (CCL2, CCL4, FGF2, GMCSF, IFNG, IL-6, IL-12p35, IL-12p70, LIF, LTA, PDGFbb, TNF and VEGF) were chosen as target candidates, and their expression in conditioned medium (n = 3 each) was assessed in 20 μg protein lysate samples along with the pre-stained molecular weight marker. Samples were assessed by SDS-PAGE and subsequent Western immunoblotting techniques on a nitrocellulose membrane and chemicals obtained from Bio-Rad (Hercules, CA, USA) [24]. The details of the antibodies used are given elsewhere (see Additional file 2: Table S2). Final visualisation was achieved using Vectastain ABC immunoperoxidase kits (Vector Laboratories, Burlingame, CA, USA). Respective primary and secondary antibody controls were run simultaneously to examine the specificity of the antibodies. The molecular weights and semi-quantitative analysis of the bands were determined using densitometric equipment (Pharos FX Plus Molecular Imager, Bio-Rad, Hercules, CA, USA) and optimised densitometric analysis software (PD Quest Advanced, Bio-Rad). The integrated measures of optical densities for individual antigens were calculated from the log of transmittance for each target antigen and normalised to total secreted protein (in micrograms).

### Quantitative real time RT-PCR

The relative expression of 17 genes (CCL2, CCL3, CCL5, CXCL10, FGF2, GCSF, GMCSF, IFNG, IL-1b, IL-6, IL-13, IL-16, IL-17, LIF, PDGFB, TNF and VEGF) selected as targets from the post-hoc analysis (as described below) of the BioPlex cytokine data was examined. All samples were assessed using glyceraldehyde 3-phosphate dehydrogenase (GAPDH) as an endogenous control and SYBR Green-based quantitative RT-PCR as described previously [27]. Briefly, total RNA was extracted using Trizol (Agilent Technologies Singapore Pvt. Ltd., Shung Avenue, Singapore), purified with DNase I (Sigma Chemical Co., St. Louis, Missouri, USA) and subjected to re-extraction when necessary. The yield and purity of the extracted RNA were verified using standard spectrophotometric methods and 1% agarose gel electrophoresis [27]. Furthermore, the RIN score of individual samples was determined using the Agilent 2100 Bioanalyzer, RNA 6000 NanoLabChip kit and Agilent 2100 Expert Software (Agilent Technologies, Inc., Santa Clara, CA, USA) [28]. Four samples that failed to yield either sufficient amounts of RNA or an acceptable RIN score (>8.0) were not included. For the real time RT-PCR, the first-strand cDNA was synthesised from 2 μg of total RNA with an optimised RevertAid™ First Strand cDNA Synthesis Kit (Fermentas, Germany), and the PCR was performed using Maxima™ SYBR Green/Fluorescein qPCR Master Mix (Fermentas, Germany) and forward and reverse primers for the respective genes.

The primers for the target genes were designed using Beacon Designer software (Labware Scientific Inc., USA) as shown in Additional file 3: Table S3 and obtained from Integrated DNA Technologies (Coralville, Iowa, USA) for target genes. The reaction was performed on a BioRad platform (iCycleriQTm Real time PCR detection system, BioRad, Hercules, CA, USA) using an optimised protocol [27]. Cycle threshold values were obtained, and ∆Ct values between the experimental and normalised Ct were determined. The relative expression ratios between groups from cycle threshold (Ct) values and absolute values were determined as described previously [27].

### Data analysis

Profiles of factors from the immunoassay data were classified into six scales depending on factor concentrations as follows (pg per microgram of protein): 0, ≤0.05; 1, >0.05 to 0.1; 2, >0.1 to 1.0; 3, >1.0 to 10.0; 4, >10.0 to 100.0; 5, >100.0. Due to individual variability, data were normalised with the mean basal values from the zero hCG group as the internal control. The quantitative values of factors in medium and transcripts in cells from each culture group with different doses of hCG were log transformed and analysed using the Kruskal-Wallis test followed by the Wilcoxon Signed Ranks test. Significant changes were derived for each bin showing P < 0.05.

### Enrichment and process networks analysis

For post-hoc enrichment analysis, candidate products were matched with known products into functional ontologies for ‘common’, ‘similar’ and ‘unique’ sets. The probability of a random intersection between a candidate on the target list and ontology entities was estimated in terms of p-values. A lower p-value meant higher relevance of the entity to the dataset due to a higher rating for the entity. Enrichment analyses were performed using a cut-off threshold (pFDR(p) = 0.05) for the cytokines, chemokines and growth factors showing differential secretion in response to rhCG to identify the enriched biological pathways. Furthermore, the uploaded files of the input list of cytokines, chemokines and growth factors showing differential secretion in response to hCG were used for the generation of biological networks. The Analyze Networks (AN) algorithm with default settings was used to retrieve interaction networks that were potentially influenced by hCG. In this workflow, the networks were prioritised based on the number of canonical pathways in the network. The enrichment analysis and network constructions were achieved using a Metacore bioinformatics platform (GeneGo, St. Joseph, MI, USA) [29].

## Results

### Characterisation of isolated cells in culture

**Figure 1:**
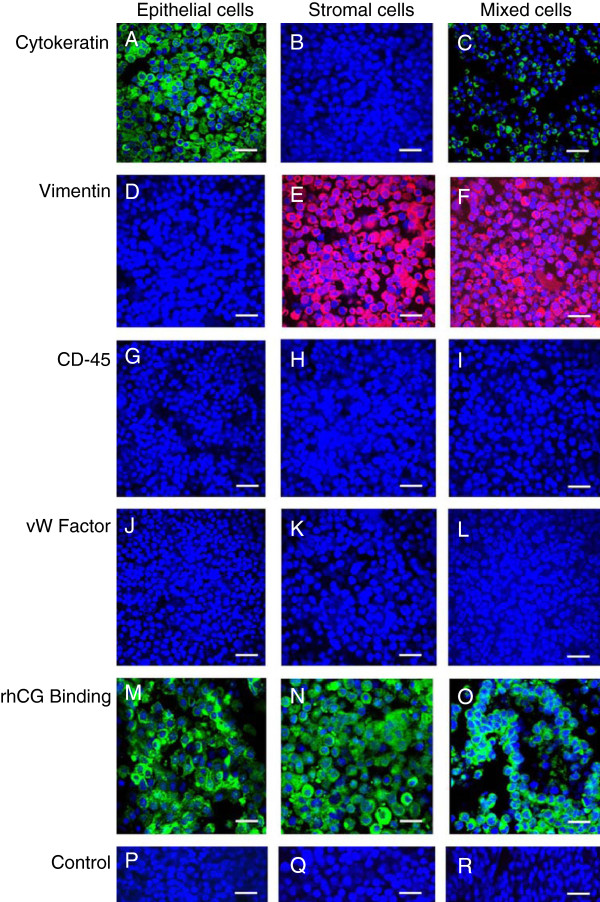
**Immunocytochemical characterisation of isolated endometrial cell populations in primary culture.** Isolated human mid-luteal phase endometrial epithelial cells **(A, D, G, J, M, P)**, stromal cells **(B, E, H, K, N, Q)** and mixed cells **(C, F, I, L, O, R)** were allowed to attach to the collagen biomatrix and subjected to immunostaining for cytokeratin (*green;***A**-**C**), vimentin (*red;***D**-**F**), CD45 (*green*; **G**-**I**), vW factor (*red*; **J**-**L**), and hCG binding (*green*; **M**-**O**) (detected with biotinylated rhCG) was determined in each cell type and in non-immune sera from mouse and goat **(P)** and non-immune sera from mouse and rabbit **(Q)** and biotinylated rhCG alone **(R)** followed by immunofluorescence detection was performed as described in the Methods section. Nuclei were counterstained with DAPI (*blue*). Bars = 50 μm.

The procedure used in the present study yielded >93% viable cells. Figure 1 (A-L) shows representative microphotographs of cytokeratin (Figure 1A-C), vimentin (Figure 1D-F), CD45 (Figure 1G-I) and vW factor (Figure 1J-L) localisation in attached epithelial, stromal and mixed cells 72 hours after seeding. The washed ‘enriched epithelial cell’ fraction yielded 88 (±2)% cytokeratin-positive epithelial cells, and the CD45-negative fraction contained the ‘enriched stromal cell’ population that yielded 92 (±4)% vimentin-positive stromal cells. Generally, there were no CD45- and vW factor-positive cells after attachment (Figure 1G-L). The endometrial mixed cells used in the study typically constituted 30 (± 4)% cytokeratin-positive epithelial cells and 70 (±4)% vimentin-positive stromal cells. Figure 1 (M-O) shows the pattern of hCG binding following exposure to biotinylated rhCG (200 mIU/ml) in epithelial cells, stromal cells and mixed cells at 72 hours in primary culture when the cells were subjected to hCG.

### General profile of secreted cytokines

The detailed profile of cytokines secreted from the three groups of cells under basal conditions (serum-free DMEM:F12 (1:1) medium supplemented with insulin (10 μg/ml), transferrin (5.5 μg/ml), selenium (6.7 ng/ml), hydrocortisone (5 μg/ml), gentamicin (10 μg/ml), penicillin (100 IU/ml), streptomycin (100 μg/ml) and fungizone (2.5 μg/ml)) is shown in the supplementary table (see Additional file 4: Table S4). In these conditions, endometrial cells in all three groups secreted more than 1 pg/μg protein levels of CCL4, CCL5, CXCL9, CXCL12, FGF2, GMCSF, HGF, IL-2ra, IL-6, IL-12p40, MCSF, MIF, and VEGF. Additionally, endometrial epithelial cells in group 1 secreted substantial amounts of CCL3, CCL7, CXCL1, CXCL10, IFNG, IL-1ra, IL-12p70, LIF, PDGFbb and SCGF; these cytokines were either not detectable or there was less than 1 pg/μg in the other two groups. CCL2 and IL-8 were secreted at >1 pg/μg of total secreted protein by endometrial stromal cells (group 2) and mixed cells (group 3) but not by endometrial epithelial cells (group 1). Both endometrial epithelial cells and stromal cells failed to secrete detectable levels of CCL27, GCSF, IL-2, IL-4, IL-7, IL-9, IL-10, IL-15, IL-18, bNGF, SCF, and LTA under basal conditions. Of these twelve cytokines, endometrial mixed cells did not show detectable levels of seven of the cytokines (CCL27, IL-10, IL-15, IL-18, bNGF, SCF, and LTA) and had negligible (>0.1 pg/μg protein) amounts of IL-2, IL-4 and IL-7; however, endometrial mixed cells secreted substantial amounts of GCSF (>10 pg/μg protein) and IL-9 (>1 pg/μg protein). Endometrial epithelial cells did not secrete detectable CCL2, and stromal cells did not secrete detectable CCL3, CCL7, IL-12p70, IL-13, PDGFbb, and TNF levels. In endometrial mixed cells, CCL7, IL-12p70 and PDGFbb were not detectable, and CCL3, IL-13 and TNF were detectable at very low concentrations (<1 pg/μg protein).

### Differential cytokine secretion profile following hCG administration

Statistical analyses failed to identify any significant change between administration of 0 IU/ml and 1 IU/ml or between 10 IU/ml and 100 IU/ml of rhCG; however, there was a significant difference between administration of 0/1 IU and 100 IU of rhCG in group 1 (epithelial cells), 2 (stromal cells) and 3 (mixed cells) cells in the production of a large number of cytokines. Table 1 gives a summary of the fold change in the concentrations of secreted cytokines as assessed by quantitative multiplex assays using conditioned media from the three groups of cells following 100 IU/ml of rhCG treatment.

**Table 1:** Fold-change (at P < 0.05) observed in secreted cytokines, chemokines and growth factors from isolated endometrial cells following administration of recombinant hCG (100 IU/ml) in three culture groups

**Name**	**Groups (Cell type)**		
	**1**	**2**	**3**
	**(Epithelial)**	**(Stromal)**	**(Mixed)**
***CCL2***^*1*^	(ι)	-	-
*CCL3*^*2*^	-	-	(+)8.0
***CCL4***	(+)1.7	(+)1.5^5^	(+)8.0
*CCL5*^*2*^	(+)1.8	-	(+)2.8
*CCL7*	(-)1.7	-	-
*CXCL1*	(-)6.5	-	-
*CXCL9*	-	-	(-)1.7
*CXCL10*^*3*^	-	-	(+)2.7
*CXCL12*	(-)2.1	-	-
***FGF2***^*3*^	-	(+)1.5	(+)2.3
*GCSF*^*2*^	-	-	(+)1.4
***GMCSF***^*3*^	-	(+)1.5	(+)2.7
*HGF*	(-)1.6	-	(-)1.7
***IFNG***^*2*^	-	(+)1.5	(+)2.0
*IL-1b*^*1*^	-	(+)2.7	(+)3.3
***IL-6***^*2*^	(+)2.1	(-)1.6	(-)2.3
*IL-8*	-	-	(-)2.6
*IL-12p40*	(-)1.5	-	-
***IL-12p70***	-	-	(ι)
*IL-13*^*2*^	-	-	(+)1.8
*IL-16*^*1*^	-	(-)2.7	(-)1.8
*IL-17*^*2*^	-	(+)2.1	-
***LIF***^*4*^	(+)1.5		(+)1.7
*MCSF*	(-)1.5	-	(-)1.6
*MIF*	(+)1.7	(-)1.5	(-)2.4
***PDGFBB***^*1*^	-	-	(ι)
*SCGF*	(-)3.5	-	-
***TNF***^*2*^	-	-	(+)1.7
*TRAIL*	(+)1.5	-	(-)1.6
***VEGF***^*1*^	-	(+)1.9	(+)2.5

_, no change. (ι), infinitesimally increased because of non-detectable amount under the basal condition. (), up-regulated. (~) down-regulation. The cytokines which were subjected to Western blot analysis are shown in *bold*, and the ones which were subjected to quantitative real time RT-PCR for estimation of relative abundance of transcripts are shown in *italics*.

^1^did not show significant concordance between immunopositive protein concentration in medium and relative abundance of transcripts for all cell culture groups (groups 1–3).

^2^showed significant (P < 0.05) concordance between immunopositive protein concentration in medium and relative abundance of transcripts for all cell culture groups (groups 1–3).

^3^showed significant (P < 0.05) concordance between immunopositive protein concentration in medium and relative abundance of transcripts for epithelial cells (group 1) and mixed cells (group 3), but not for stromal cells (group 2).

^4^showed significant (P < 0.05) concordance between immunopositive protein concentration in medium and relative abundance of transcripts for stromal cells (group 2) and mixed cells (group 3), but not for epithelial cells (group 1).

^5^no concordance was seen between profiles from multiplex based immunoassay and from densitometric analysis of Western immunoblot.

### Immunochemical and transcript analyses

**Figure 2:**
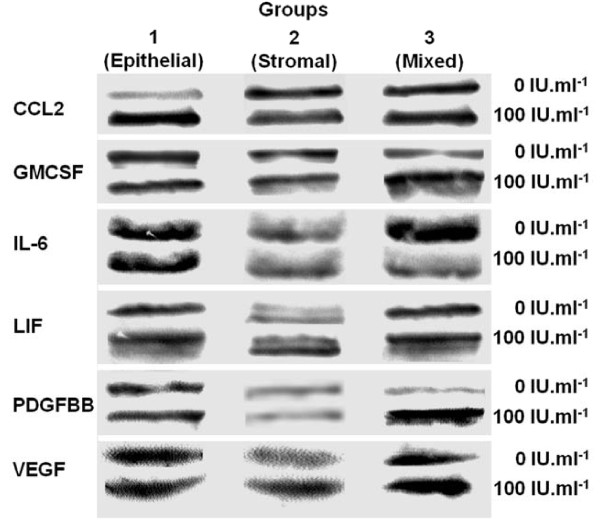
**Representative Western blot analysis of selected cytokines.** Immunopositive images of CCL2, GMCSF, IL-6, LIF, PDGFBB and VEGF in conditioned media with isolated human endometrial epithelial cells (Group 1), stromal cells (Group 2) and mixed cells (Group 3) treated without or with rhCG (100 IU/ml) are shown. Conditioned media from different groups (20 μg of protein determined by Bradford assay) were subjected to electrophoretic separation followed by immunoblot analysis. The relative optical densities were measured by integrated image analysis per μg of protein.

Marked concordance was observed between estimates of changes in concentrations obtained from multiplex immunoassays and from Western blot analyses for thirteen cytokines. An exception was CCL4 in the conditioned media of stromal cells in which a positive significant change following 100 IU of hCG administration was demonstrated in multiplex assays, but no detectable change was observed in the Western immunoblot analysis (Table 1). Figure 2 shows representative Western blot images for the cytokines with significant changes in expression (P < 0.05).

**Figure 3:**
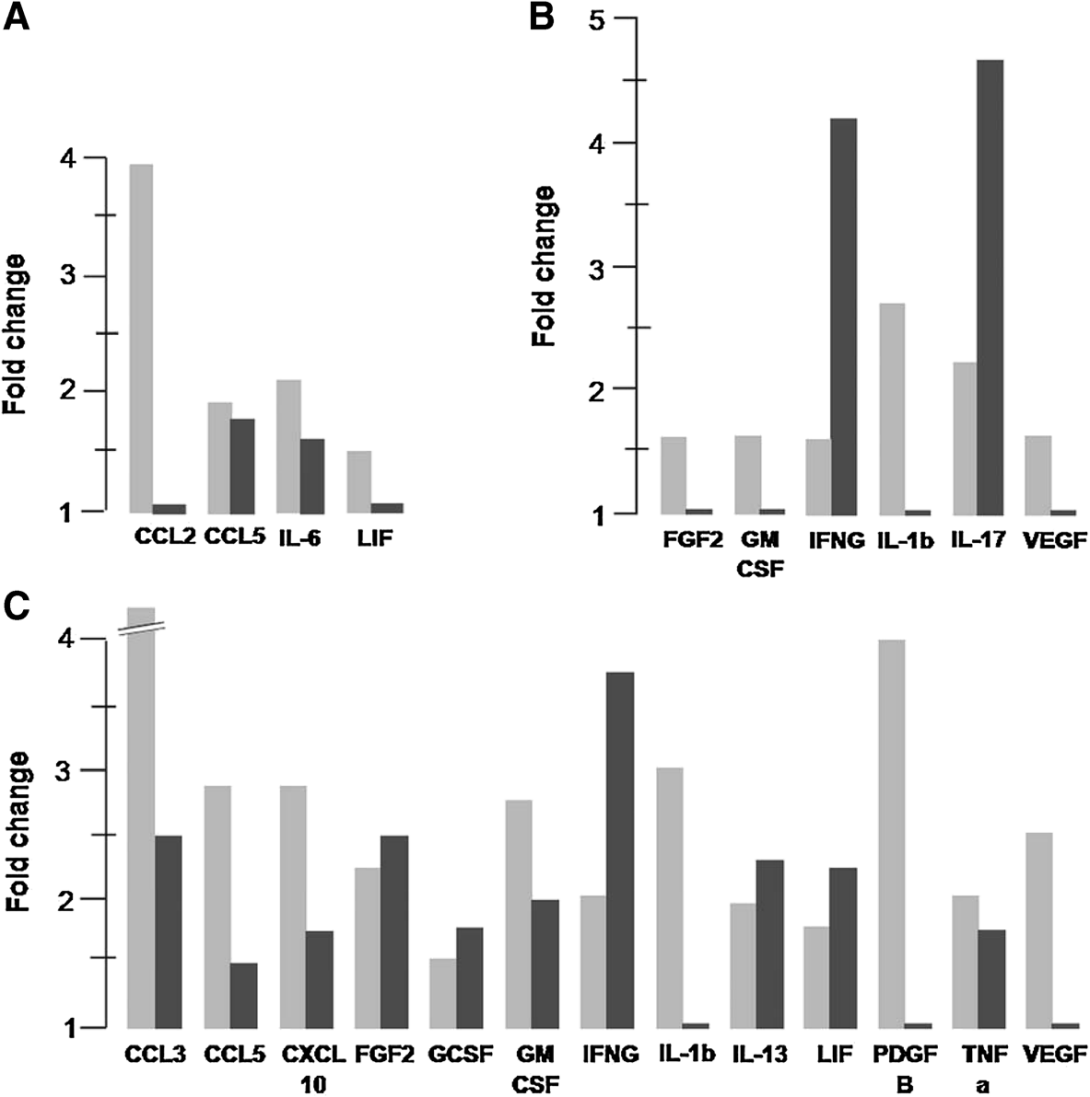
**Bar diagram showing fold increase in protein and transcript levels from cells treated *in vitro *with and without hCG.** Fold increase in protein levels between cultures treated without or with rhCG (100 IU/ml) in conditioned media (*grey bar*) and relative abundance of transcripts (*black bar*) in cultured **(A)** endometrial epithelial cells, **(B)** endometrial stromal cells, and **(C)** endometrial mixed cells are shown. The pattern of regulation by hCG depending on cell type and cytokine is evident. For example, the protein level of CCL2 is high but shows very little change at the transcript level, whereas levels of IL-6 at both the protein and transcript level are high following rhCG treatment only in epithelial cells **(A)**. Similarly, the protein levels of FGF2 and GMCSF are high but show very little change at the transcript level in stromal cells **(B)**, whereas both protein and transcript levels are high in the mixed cells **(C)**; these cytokines were unaffected in epithelial cells following hCG treatment. The pattern of changes in IFNG gene expression and protein secretion is very similar between endometrial stromal cells **(B)** and endometrial mixed cells **(C)** following administration of hCG.

On the other hand, the expression of seventeen genes based on quantitative real time RT-PCR revealed marked variation between changes in protein secretory profiles and the relative abundance of transcripts in the cells following treatment with rhCG (100 IU/ml). Figure 3 shows a graphical comparison of the changes observed in cytokine protein levels in the conditioned media and in the relative abundance of transcripts of these cytokines, chemokines and growth factors in the cultured cells; the data demonstrated a significant increase in the cytokine transcript levels following treatment with 100 IU rhCG compared with that in the basal condition.

### Enrichment analysis and biological networks construction

Table 2 shows the summary of the enrichment analysis for the candidate cytokines, chemokines and growth factors showing differential (≥1.5) secretion at P < 0.05 following the administration of hCG in all three cell culture groups. The integral modules that were affected by hCG treatment were immune response, chemotaxis, inflammatory changes, proliferation, cell adhesion and anti-apoptosis. It is notable that the hierarchical analysis of pathways and GO processes indicated a differential behaviour among epithelial, stromal and mixed cells upon treatment with hCG (Table 2).

**Table 2:** Summary of top scored features retrieved from reports of enrichment analysis from input list of cytokines, chemokines and growth factors showing high basal secretion and/or differential secretion following administration of hCG

**Enrichment by**	**Types of cell culture**		
	**Epithelial cells**	**Stromal cells**	**Mixed cells**
	**(Active products)**	**(Active products)**	**(Active products)**
	**[pValue; FDR]**	**[pValue; FDR]**	**[pValue; FDR]**
***Pathway maps***	**Immune response**	**Immune response**	**Immune response**
	(CCL2, CCL4, CCL5, CCL7, XCL1, IL-6, IL-12, HGF)	(CCL4, GMCSF, IFNG, IL-1b, IL-6, IL-17, MIF, VEGF)	(CCL3, CCL4, CCL5, GCSF, GMCSF, HGF, IFNG, IL-1b, IL-6, MIF, TNF, VEGF)
	[8.3E-08; 1.6E-05]	[8.7E-08; 8.5E-06]	[1.9E-09; 4.3E-07]
	**Macropinocytotic transport**		**Epithelial-mesenchymal transition**
	(HGF, MCSF)		(FGF2, HGF, IL-1b, TNF)
	[1.2E-05; 5.7E-04]		[7.9E-05; 1.7E-05]
***GO process***	**Chemotaxis, leukocyte chemotaxis**	**Chemotaxis, leukocyte chemotaxis**	**Regulation of response to stimulus, signal transduction and cell communication**
	(CCL2, CCL3, CCL4, CCL5, CCL7, CXCL1, CXCL12, HGF, IL-6, MIF)	(CCL4, IFNG, IL-1b, IL-6, IFNG, MIF, VEGF)	(CCL4, CCL5, CXCL12, FGF2, GCSF, GMCSF, HGF, IL-13, LIF, MCSF, MIF, TNF, VEGF)
	[9.9E-21; 7.9E-18]	[2.0E-15; 3.2E-12]	[1.8E-27; 3.7E-24]
		**Regulation of response to stimulus**	**Chemotaxis, leukocyte chemotaxis**
		(CCL4, FGF2, IL-1b, IL-6, IL-17 VEGF)	(CCL3, CCL4, CCL5, CXCL12, FGF2, HGF, IFNG, IL-1b, IL-6, MIF)
		[8.4E-14; 4.6E-11]	[5.2E-22; 2.8E-19]
			**Immune response**
			(CCL3, CCL4, CCL5, CXCL9, CXCL10, GCSF, MCSF, IFNG, IL-1b, IL-6, IL-13, LIF, MCSF, MIF, TNF, VEGF)
			[4.0E-21; 1.2E-18]
***Process networks***	**Inflammation**	**Immune response**	**Inflammation**
	(CCL2, CCL3, CCL4, CCL5, CCL7, CXCL12, HGF, IL-6, IL-12, LIF, MCSF)	(GMCSF, IL-1b, IL-6, IL-17, MIF)	(CCL3, CCL5, GCSF, GMCSF, IFNG, IL-6, IL-13, LIF, MCSF, TNF)
	[2.9E-09; 2.4E-07]	[4.9E-09; 3.2E-07]	[9.6E-13; 9.3E-11]
	**Chemotaxis**	**Signal transduction**	
	(CCL2, CCL3, CCL4, CCL5, CCL7, CXCL1, CXCL12)	(GMCSF, IFNG, IL-1b, VEGF)	
	[5.7E-09; 2.4E-07]	[9.9E-06; 3.1E-04]	
	**Chemotaxis**	**Signal transduction**	**Proliferation**
	(CCL2, CCL3, CCL4, CCL5, CCL7, CXCL1, CXCL12)	(GMCSF, IFNG, IL-1b, VEGF)	(CXCL12, FGF2, GCSF, GMCSF, HGF, LIF, VEGF)
	[5.7E-09; 2.4E-07]	[9.9E-06; 3.1E-04]	[6.6E-12; 3.2E-10]
	**Immune response**	**Inflammation**	**Cell adhesion**
	(CXCL12, CCL2, CXCL1, IL-6, CCL7, MIF)	(IL-1b, GMCSF, IFNG, CCL4, IL-6)	(IL-1b, TNF, CCL3, VEGF, MIF, FGF2, CCL5, CCL4, CXCL12)
	[1.5E-08; 2.4E-07]	[1.5E-06; 3.1E-04]	[3.3E-10; 1.1E-08]
	**Cell adhesion**	**Anti-apoptosis**	**Chemotaxis**
	(CCL2, CCL3, CCL4, CCL5, CCL7, CXCL1, CXCL12)	(FGF2, GMCSF, IL-1b, IL-6, VEGF)	(CCL3, CCL4, CCL5, CXC12, FGF2, IL-1b, MIF, VEGF)
	[1.4E-08; 2.9E-06]	[2.3E-05; 3.7E-04]	[9.4E-10; 2.3E-08]
	**Proliferation**	**Cell adhesion**	**Anti-apoptosis**
	(CCL4, CCL5, CXCL12, HGF, IL-12)	(FGF2, IL-1b, IL-6, VEGF)	(CCL3, FGF2, GMCSF, HGF, IL-1b, IL-6, TNF, VEGF)
	[2.4E-07; 4.1E-06]	[1.4E-04; 1.0E-03]	[5.8E-09; 9.4E-08]
			**Immune response**
			(GCSF, GMCSF, IL-1b, IL-6, MIF)
			[5.2E-08; 6.3E-07]

*FDR* false discovery rate.

## Discussion

The cells in the three culture groups showed differential profiles of cytokine, chemokine and growth factor secretion in the present study. Table 3 provides a summary of the unique secretory profiles observed in the different experimental groups. It appears that the multiplexed method of simultaneous immunoassay analysis is robust, as the profiles generally concurred well with the profiles determined by Western blot analysis. The observed mismatch in cytokines was probably due to differences either in sensitivities (CCL2, PDGFbb) or target epitopes (IL-1b and VEGF) between the two immunochemical methods. Furthermore, the three-dimensional primary culture model in conjunction with large-scale assays is a robust model system to study the endocrine, paracrine and juxtacrine aspects of the complex regulation of implantation-stage endometrial cells as suggested by other groups [30,31].

**Table 3:** Summary of the major observations from the present study

**(Description) Group**	**Concentration level of secretion (pg in μg protein) in basal condition**	**Name of cytokines showing differential**^**1 **^**secretion following administration of hCG**
Group 1	≤1	**CCL2** (+), **TRAIL** (+)
(Endometrial epithelial cells)	>1-10	CCL5 (+), **CCL7** (-), **CXCL12** (-), HGF (-), **IL-12p40** (-), LIF (+), MCSF (-)
	>10-100	CCL4 (+), **CXCL1** (-), **MIF** (+), **SCGF** (-)
	>100	**IL-6** (+)
Group 2	≤1	IL1b (+), IL-16 (-)**, IL17** (+)
(Endometrial stromal cells)		CCL4 (+), FGF2 (+), IFNG (+), IL-6 (-), VEGF (+)
	>1-10	GMCSF (+), MIF (-)
	>10-100	None
	>100	
Group 3	≤1	**CCL3** (+), **CXCL10** (+), IL-1b (+), **IL-12p70** (+), **IL-13** (+), IL-16 (-), LIF (+), **PDGFbb** (+), **TNF** (+), **TRAIL** (-)
(Endometrial mixed cells)	>1-10	CCL4 (+), CCL5 (+), **CXCL9** (-), FGF2 (+), GMCSF (+), HGF (-), IFNG (+), MCSF (-)
	>10-100	**GCSF** (+), **IL-8** (-), MIF (-), VEGF (+)
	>100	IL-6 (-)

(+), up-regulated. (-), down-regulated.

The cytokines, chemokines and growth factors which were regulated in cell culture group specific manner are shown in *bold*.

^1^≥ 1.5-fold at P < 0.05.

### Cell type specificity of secretory profiles under basal conditions

Although CCL4, CCL5, CXCL9, CXCL12, FGF2, GMCSF, HGF, IL-2ra, IL-6, IL-12p40, MCSF, MIF and VEGF were observed to be consistently produced by all cells types, cell type specificity without the addition of hCG was evident. Endometrial epithelial cells showed a markedly different secretory profiles as compared with stromal cells; for example, the concentrations of CCL3, CCL7, CXCL1, CXCL10, IFNG, IL-1ra, IL-12p70, LIF, PDGFbb, and SCGF were much higher in the conditioned media of endometrial epithelial cells as compared with that of stromal cells, whereas the concentrations of CCL2 and IL-8 were much higher in the conditioned media of endometrial stromal cells as compared with that of epithelial cells. Clearly, epithelial cells secreted multiple cytokines.

As expected, there were marked similarities in the secretory profiles of most of the cytokines, chemokines and growth factors between homologous endometrial epithelial cells or stromal cells and heterologous mixed cells comprised of endometrial epithelial (≈30%) plus stromal cells (≈70%). However, two mediators showed differential profiles, GCSF and IL-9; although these factors were absent in the conditioned media of both endometrial epithelial cells and stromal cells, they were secreted in substantial amounts by mixed cells in heterologous culture. A high level of GCSF secretion only by mixed cells appears to be an interesting combinatorial paracrine function of the endometrium in view of the reported positive action of GCSF on endometrial stromal cells and growth for embryo implantation [32,33]. Many of the cytokines that were secreted by endometrial cells in basal culture conditions are known to be present in human endometrial cells and uterine fluid and directly and indirectly influence endometrial receptivity and embryo implantation [14,34-38].

### Cell type-specific differential responses to hCG

Many of the cytokines, chemokines and growth factors were differentially secreted in the medium following administration of recombinant hCG (100 IU/ml), especially from endometrial epithelial cells and mixed endometrial cells. It is notable that an hCG level of 100 IU/ml appears in the circulation during the first 7 weeks of pregnancy and that there is no allometric algorithm to determine the local level of hCG; thus, a significant modulatory effect of intraluminal administration of hCG could be recorded only at 500 IU/ml [10,39]. Furthermore, the fact that there is a discernable difference in the cellular physiology of *in vivo* versus *in vitro* mammalian cells is well acknowledged [19,22,23]. Thus, we believe that the dosage for effective hCG (100 IU/ml) action may be specific to the *in-vitro* experimental model.

Many of the cytokines, chemokines and growth factors that were affected by hCG in the present study have also been reported to be influenced by hCG in the human endometrium in different experimental models as shown in Table 4. On the other hand, to our knowledge, this is the first report identifying the increased secretion of CCL3 (MIP-1a), CCL4 (MIP-1b), CCL5 (RANTES), IFNG, IL-13, PDGFbb and TRAIL and the decreased secretion of CCL7 (MCP-3), CXCL1 (GRO-a), CXCL9 (MIG), CXCL12 (SDF-1), HGF (SOS), IL-8, IL-16 and SCGF by mid-luteal phase endometrial cells following the *in vitro* administration of hCG. However, there are several reports indicating that many of these cytokines, chemokines and growth factors in implantation-stage endometrium may be regulated by various paracrine mediators, such as INFG, IL-1, IL-6, PAF, thrombin and TNF, which are potentially secreted from implantation-stage endometrium and the embryo [40-52]. We observed that hCG could influence the secretion of many of these paracrine mediators (INFG, IL-1b, IL-6, and TNF) by implantation-stage endometrium. Furthermore, some of the cytokines, chemokines and growth factors (CCL3, CCL4, CCL5, FGF2, GCSF, GMCSF, IL-1b, IL-6, IL-8, LIF, PDGFbb, TNF and VEGF) that were found to be differentially regulated by hCG in endometrial cells are known to have direct or indirect action in supporting embryo implantation [33,53-55], and indeed a few (CCL2, CXCL10, IL-1b and TNF) have shown significant correlation with successful implantation and establishment of clinical pregnancy [56].

**Table 4:** A summary of the results from the present study and previous reports for common endometrial secretions observed to be affected by hCG

**Name**	**Observation in the present study; affected cell culture type**	**Observation in the previous studies; experimental model [Reference]**	**Putative function in implantation process [Reference]**
CCL2	Increase: epithelial HEnC	No change; mixed endometrial cells^1^[45]	Regulation of angiogenic and immunotolerant environment [57,58]
CXCL10	Increase; mixed HEnC	Increase; HEEC^1^[14]	Regulation of NK cell recruitment and angiogenesis [59,60]
FGF2	Increase; stromal HEnC and mixed HEnC	Increase; HEEC^1^[14]	Regulates endometrial stromal cell proliferation and receptivity and embryo growth, attachment and trophoblast function [61,62]
GMCSF	Increase; stromal HEnC and mixed HEnC	Increase; HEEC^1^[14]	Regulates endometrial stromal cells, leukocytes and capillaries along with embryotropic action [34,53,63]
IL-1b	Increase; stromal HEnC and mixed HEnC	Increase; intrauterine secretion^2^[39]	Mediates endometrial preparation for implantation [36,58]
		No change; mixed endometrial cells^1^[45]	
		Increase; endometrial secretion aspiration^3^[64]	
IL-6	Increase; epithelial HEnC Decrease; stromal HEnC and mixed HEnC	Increase; intrauterine secretion^2^[39]	Regulates hCG secretion by trophoblast cells and endometrial development [36,65,66]
		Increase; mixed endometrial cells^1^[67]	
		Decrease; HEEC^1^[7]	
		No change; HEEC^1^[14]	
IL-12	Decrease (p40); epithelial HEnC	Endometrial secretion aspiration^3^[64]	Regulates NK cell function and mediates cell mediated immunity [68]
	Increase (p70); mixed HEnC		
IL-17	Increase; stromal HEnC	Increase; endometrial secretion aspiration^3^[64]	Potentially regulates chemokines and cytokines networks [69]
LIF	Increase; epithelial HEnC and mixed HEnC	No change; mixed endometrial cells^1^[67]	Supports endometrial receptivity and embryo development [36]
		Increase; intrauterine secretion^2^[39]	
		Increase; HEEC^1^[7,14]	
MCSF	Decrease; epithelial HEnC and mixed HEnC	Decrease; intrauterine secretion^2^[39]	Paracrine regulation of stromal decidualization under progesterone dominance [70,71]
MIF	Increase; epithelial HEnC and mixed HEnC	Increase; endomerial stromal cells^1^[9]	Paracrine regulation of immunomodulation, angiogenesis and growth [47,72]
	Decrease; stromal HEnC		
TNF	Increase; mixed HEnC	Increase; mixed endometrial cells^1^[64]	Regulates balance of cytokine, chemokines and growth factors towards endometrial and embryo development [73,74]
		Increase; endometrial secretion aspiration^3^[39]	
VEGF	Increase; stromal HEnC and mixed HEnC	Increase; intrauterine secretion^2^[39]	Regulates endometrial angiogenesis, permeability receptivity and embryo development [36,75,76]
		Increase; HEEC^1^[7,14,77]	
		Increase; mixed endometrial cells^1^[78]	
		Increase; epithelial HEnC [25]	

HEnC, endometrial cells in primary culture on collagen biomatrix. HEEC, isolated human endometrial epithelial cells. HEExC, human endometrial explant culture.

^1^Conventional primary culture.

^2^Intrauterine microdialysis. ^3^endometrial secretion aspired on the day of embryo transfer in stimulated cycle in IVF-ET and compared with that in natural cycle.

### CG may control the endometrial informational networks that promote receptivity

**Figure 4:**
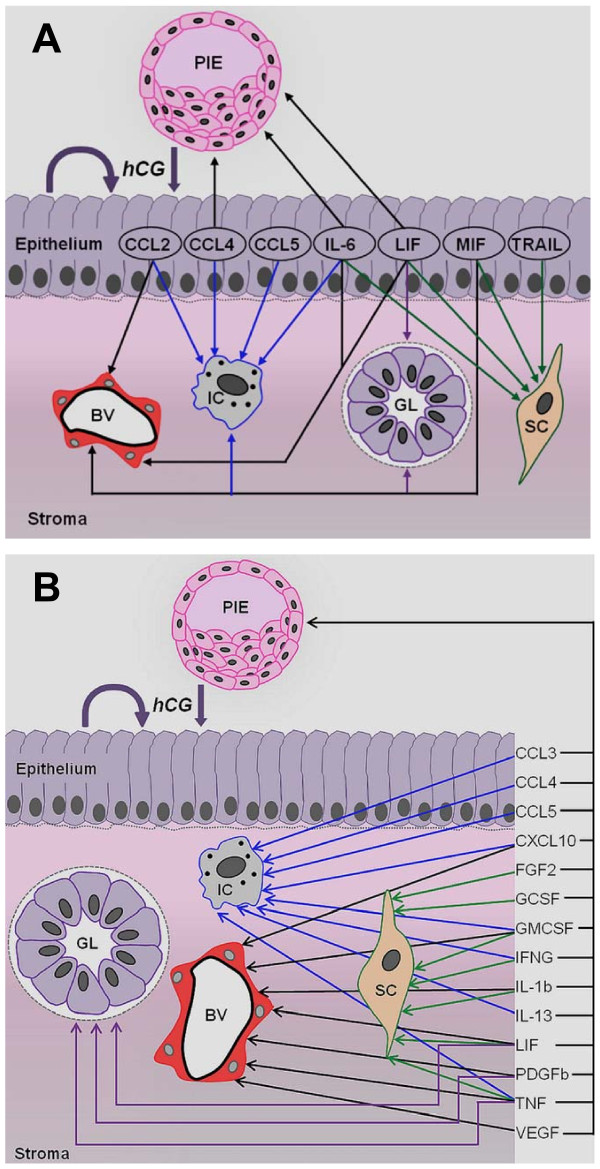
**A proposed model of the paracrine action mediated by hCG that involves different cell types in the endometrium and that affects the implantation-stage embryo and endometrium.** The pre-implantation embryo- and endometrium-derived CG may specifically act on endometrial surface epithelium alone **(A)** or may act on both the endometrial epithelial cells and stromal fibroblasts **(B)**, resulting in up-regulation of a specific cohort of cytokines, chemokines and growth factors. Many of these factors (e.g., CCL3, CCL4, CCL5, CXCL10, FGF2, GMCSF, IFNG, IL-1b, IL-6, IL-13, LIF, PDGFb, TNF and VEGF) are reportedly embryotropic, some (e.g., IL-6 and LIF) are primarily secreted by endometrial epithelial cells, and a few (e.g., FGF2, GMCSF, INFG, IL-1b and VEGF) are secreted primarily by endometrial fibroblasts in response to hCG. In contrast, many of the secreted factors are known to regulate stromal fibroblasts (e.g., FGF2, GCSF, GMCSF, IFNG, IL-1b, IL-6, LIF, MIF, TNF and TRAIL), vascular physiology (e.g., CCL2, CXCL10, GMCSF, IL-1b, IL-6, LIF, MIF, PDGFb, TNF and VEGF) and glandular physiology (e.g., LIF, MIF, PDGFb, and TNF). Only a few of the factors (e.g., CCL2, IL-6, LIF and MIF) are secreted by isolated endometrial epithelial cells in response to hCG. Interestingly, many of these factors directly and indirectly regulate the functions of immunocompetent cells. A large number of reports [21,32-53,57-78,80] and the results of the present study have been used to develop this model. BV (blood vessel), FB (fibroblast), GL (gland), IC (immunocompetent cells), PIE (pre-implantation embryo). There is no quantitative aspect to the length and thickness of arrows.

The results obtained in the present study strongly corroborate the notion that hCG influences the physiology of the endometrium and embryo during the time of blastocyst implantation. Based on a large number of previous reports [21,32-80], we have constructed a tentative model of the hCG-mediated paracrine activity controlling implantation of the embryo and the endometrium (Figure 4). It is apparent that several embryotropic cytokines, chemokines and growth factors, which include CCL3, CCL4, CCL5, CXCL10, FGF2, GMCSF, IFNG, IL-1b, IL-6, IL-13, LIF, PDGF, TNF and VEGF, were up-regulated in implantation-stage endometrium by hCG. Furthermore, several of these cytokines are known to regulate stromal fibroblasts (e.g., FGF2, GCSF, GMCSF, IFNG, IL-1b, IL-6, LIF, MIF, TNF and TRAIL), vascular physiology (e.g., CCL2, CXCL10, GMCSF, IL-1b, IL-6, LIF, MIF, PDGFbb, TNF and VEGF) and glandular physiology (e.g., LIF, MIF, PDGFbb, and TNF). Many of these factors directly and indirectly regulate the function of immunocompetent cells. Thus, CG may affect endometrial functions related to inflammation, differentiation, proliferation, apoptosis, immune surveillance and immune tolerance through the production of a set of cytokines, chemokines and growth factors during the time of blastocyst implantation. Additionally, hCG influences macropinocytic transport in epithelial cells, regulates responses to external stimuli in stromal cells and promotes epithelial-mesenchymal transition in mixed cells. These are well-known biological features of implantation-stage endometrial tissue [79]. Further networks analysis in determining the hierarchy of different functional processes revealed that the regulation of inflammation, proliferation, cell adhesion, chemotaxis, apoptosis and immune response are the dominant actions of hCG on implantation-stage endometrium.

In general, administration of CG can influence the function of the endometrium during embryo implantation. There are at least three aspects of CG-regulated endometrial informational networks that may further be examined to elucidate its potential involvement in the implantation process. First, studies are required to understand the mechanism by which CG regulates the above-mentioned physiological modules, namely inflammation, proliferation, cell adhesion, chemotaxis, apoptosis and immune response, on the background of progesterone dominance in endometrial cells during implantation. Secondly, further studies are necessary to unravel how CG could exert differential effects on transcript and protein levels at the steady state depending on cell type and cytokine as revealed in the present study. A large number of reports indicate that manifestation of such poor correlations suggests multi-level regulation by an input signal [81-84], which was hCG in this study. Thirdly, additional multi-scale and multi-level studies are necessary to understand the CG-mediated regulation of endometrial functions at implantation that involve paracrine control between cell types.

## Conclusions

Several interesting points may be concluded from the results of the present study. First, the primary cell culture of human endometrial cells on collagen biomatrix is a robust experimental model to study the endocrine, paracrine and juxtacrine actions of a biological molecule, hCG. Despite the fact that there were several cytokines, chemokines and growth factors commonly secreted by isolated endometrial epithelial cells, stromal cells and mixed cells under basal conditions, there were many cytokines that were secreted specifically by endometrial epithelial cells. However, a substantial level of GCSF was produced by endometrial cells only when epithelial and stromal cellular elements were mutually interactive. Thus, GCSF appears to be a potential physiological marker of the functional integrity of the endometrium. Furthermore, it was demonstrated for the first time that administration of hCG could affect isolated endometrial epithelial cells, stromal cells and mixed cells in a differential fashion. Many of the factors are known to exert paracrine influence on implantation-stage endometrium (e.g., IFNG, IL-1b, IL-6 and TNF) and support embryo implantation (e.g., CCL3, CCL4, CCL5, FGF2, GCSF, IL-1b, IL-6, IL-8, LIF, PDGFbb, TNF and VEGF) through their regulatory actions on inflammation, proliferation, cell adhesion, chemotaxis, apoptosis and immune responses during blastocyst implantation. It is thus apparent that embryo- and endometrial-derived CG can influence implantation-stage endometrial functions through complex processes involving various cell types in the endometrium. Finally, this study provided a panel of specific cytokines, chemokines and growth factors that are secreted by various cell types in the endometrium during the window of implantation.

## Competing interests

The authors declare that they have no competing interests.

## Authors’ contributions

AKS contributed in performing experiments, data acquisition and data analysis. JS and DG contributed to the study design, acquisition, analysis and interpretation of data, and drafting of the manuscript. AK and KKR contributed in providing human samples and data interpretation. All authors read and approved the final manuscript.

## Supplementary Material

Additional file 1: Table S1 Cytokines, chemokines and growth factors studied. Click here for file

Additional file 2: Table S2 Primary antibodies used in the study. Click here for file

Additional file 3: Table S3 List of genes and their primers used for quantitative real time RT-PCR. Click here for file

Additional file 4: Table S4 Profiles of secreted cytokines, chemokines and growth factors from isolated endometrial cells of three culture groups in the basal condition. Click here for file
