# Supplementary material for: Profiles of cytokines secreted by isolated human endometrial cells under the influence of chorionic gonadotropin during the window of embryo implantation
Source: Reprod Biol Endocrinol. 2013 Dec 17;11:116. doi: 10.1186/1477-7827-11-116 (PMC3878507; doi:10.1186/1477-7827-11-116)
Supplement: Additional file 1: Table S1 — Cytokines, chemokines and growth factors studied. [file 1477-7827-11-116-S1.doc]

**Additional file 1: Table S1 Cytokines, chemokines and growth factors studied**

___________________________________________________________________________

Name Full name Alternative/former name

___________________________________________________________________________

CCL2 Chemokine (C-C Motif) Monocyte Chemoattractant Protein 1

Ligand 2 (MCP-1)

CCL3 Chemokine (C-C Motif) Macrophage Inflammatory Protein 1-alpha

Ligand 3 (MIP-1alpha)

CCL4 Chemokine (C-C Motif) Macrophage Inflammatory Protein 1-beta

Ligand 4 (MIP-1beta)

CCL5 Chemokine (C-C Motif) Regulated Upon Activation, Normally T-

Ligand 5 Expressed, and Presumably Secreted (RANTES)

CCL7 Chemokine (C-C Motif) Monocyte Chemoattractant Protein 3

Ligand 7 (MCP-3)

CCL11 Chemokine (C-C Motif) Eosinophil Chemotactic Protein

Ligand 11 (Eotaxin-1)

CCL27 Chemokine (C-C Motif) Cutaneous T-cell-Attracting Chemokine

Ligand 27 (CTACK)

CXCL1 Chemokine (C-X-C Motif) Growth-Regulated alpha Protein

Ligand 1 (GRO-alpha)

CXCL9 Chemokine (C-X-C Motif) Monokine Induced by Interferon-Gamma

Ligand 9 (MIG)

CXCL10 Chemokine (C-X-C Motif) Interferon Gamma-Induced Protein

Ligand 10 (IP-10)

CXCL12 Chemokine (C-X-C Motif) Stromal Cell-Derived Factor 1

Ligand 12 (SDF-1)

FGF2 Fibroblast Growth Factor 2 Basic Fibroblast Growth Factor

(bFGF)

GCSF Granulocyte Colony-Stimulating Colony Stimulating Factor 3

Factor (CSF-3)

GMCSF Granulocyte-Macrophage Colony Colony Stimulating Factor 2

-Stimulating Factor (CSF-2)

HGF Hepatocyte Growth Factor Hepatocyte Growth Factor

(HGF)

IFNa2 Interferon, alpha 2 Interferon alpha-A (IFN-alphaA)

IFNG Interferon, gamma Immune Interferon (IFI)

IL-1a Interleukin-1, alpha Hematopoietin-1

IL-1b Interleukin 1, beta Catabolin

IL-1ra Interleukin 1 Receptor Antagonist Interleukin 1 Receptor Antagonist

(IL1RN)

IL-2 Interleukin 2 T-Cell Growth Factor (TCGF)

IL-2ra Interleukin 2 Receptor, alpha T-Cell Growth Factor receptor

(TCGFR)

IL-3 Interleukin 3 Multipotential Colony-Stimulating Factor

(MULTI-CSF)

IL-4 Interleukin 4 B-Cell Growth Factor 1 (BCGF-1)

IL-5 Interleukin 5 Eosinophil Differentiation Factor

(EDF)

IL-6 Interleukin 6 Interferon beta-2 (IFNB2)

IL-7 Interleukin 7 Interleukin 7

IL-8 Interleukin 8 Chemokine (C-X-C Motif) Ligand 8

(CXCL8)

IL-9 Interleukin 9 T-Cell Growth Factor p40 (HP40)

IL-10 Interleukin 10 T-Cell Growth Inhibitory Factor (TGIF)

IL-12p40 Interleukin 12 Subunit p40 Interleukin-12 subunit beta (IL12-beta)

IL-12p70 Interleukin 12 (Holo) Natural Killer Cell Stimulatory Factor

(NKSF)

IL-13 Interleukin 13 Interleukin 13

IL-15 Interleukin 15 Interleukin 15

IL-16 Interleukin 16 Lymphocyte Chemoattractant Factor

(LCF)

IL-17 Interleukin 17 Cytotoxic T-Lymphocyte-Associated

Antigen 8 (CTLA8)

IL-18 Interleukin 18 Interferon Gamma-Inducing Factor

(IGIF)

LIF Leukemia Inhibitory Factor Differentiation Inhibitory Activity

(DIA)

LTA Lymphotoxin alpha Tumor Necrosis Factor Beta

(TNF-beta)

MCSF Macrophage Colony-Stimulating Colony-Stimulating Factor 1 Factor (CSF1)

MIF Macrophage Migration Inhibitory Glycosylation-Inhibiting Factor

Factor (GIF)

bNGF beta-Nerve Growth Factor Nerve Growth Factor, Beta Subunit

(NGFB)

PDGFbb Platelet-Derived Growth Factor Platelet-Derived Growth Factor 2

Beta Polypeptide (PDGF2)

SCF Stem Cell Factor c-Kit ligand (c-KL)

SCGF Stem Cell Growth Factor Lymphocyte Secreted Long form

of C-type Lectin (LSLCL)

TNF  Tumor Necrosis Factor Tumor Necrosis Factor-alpha

(TNF-alpha)

TRAIL TNF-Related Apoptosis Inducing Tumor Necrosis Factor (Ligand)

Ligand Superfamily, Member 10 (TNFSF10)

VEGF Vascular Endothelial Growth Vascular Endothelial Growth Factor A

Factor (VEGFA)

___________________________________________________________________________
