# Supplementary material for: Profiles of cytokines secreted by isolated human endometrial cells under the influence of chorionic gonadotropin during the window of embryo implantation
Source: Reprod Biol Endocrinol. 2013 Dec 17;11:116. doi: 10.1186/1477-7827-11-116 (PMC3878507; doi:10.1186/1477-7827-11-116)
Supplement: Additional file 2: Table S2 — Primary antibodies used in the study. [file 1477-7827-11-116-S2.doc]

**Additional file 2: Table S2 Primary antibodies used in the study**

_______________________________________________________________________

Antigen Specification of Concentration Purpose

antibody

_______________________________________________________________________

Biotin Mouse monoclonal^c^ 1 μg/ml ICC

CD45 Mouse monoclonal^a^ 1:100 ICC

CCL2 Mouse monoclonal^d^ 0.4 μg/ml WB

CCL4 Goat polyclonal^d^ 0.4 μg/ml WB

Cytokeratin Pan Mouse monoclonal^a^ 0.4 μg/ml ICC

FGF2 Goat polyclonal^d^ 0.4 μg/ml WB

GMCSF Rabbit polyclonal^d^ 0.4 μg/ml WB

IFNG Goat polyclonal^d^ 0.4 μg/ml WB

IL-6 Mouse monoclonal^d^ 0.4 μg/ml WB

IL-12 Rabbit polyclonal, p35^d^ 0.4 μg/ml WB

Goat polyclonal, p40^d^ 0.4 μg/ml WB

LIF Goat polyclonal^e^ 0.1 μg/ml WB

LTA^1^ Goat polyclonal^e^ 1.5 μg/ml WB

PDGFB Rabbit polyclonal^d^ 0.4 μg/ml WB

TNF^2^ Mouse monoclonal^d^ 0.4 μg/ml WB

VEGF Mouse monoclonal^f^ 20 μg/ml WB

Vimentin Goat polyclonal^b^ 1:20 ICC

vW factor Rabbit polyclonal^a^ 1:200 ICC

_______________________________________________________________________

ICC, immunocytochemistry. WB, Western blot analysis. ^a^Dako, Glostrup, Denmark. ^b^Sigma Aldrich, St Louis, MO, USA. ^c^Roche Diagnostics, Germany. ^d^Santa Cruz Biotechnology, Santa Cruz, CA, USA. ^e^R&D Systems, Minneapolis, MN, USA. ^f^Thermo Fisher Scientific Inc, Campus Drive, Kalamazoo, MI, USA. ^1^used as a negative control.
