# Supplementary material for: Profiles of cytokines secreted by isolated human endometrial cells under the influence of chorionic gonadotropin during the window of embryo implantation
Source: Reprod Biol Endocrinol. 2013 Dec 17;11:116. doi: 10.1186/1477-7827-11-116 (PMC3878507; doi:10.1186/1477-7827-11-116)
Supplement: Additional file 3: Table S3 — List of genes and their primers used for quantitative real time RT-PCR. [file 1477-7827-11-116-S3.doc]

**Additional file 3: Table S3 List of genes and their primers used for quantitative real time RT-PCR**

______________________________________________________________________

Serial Gene Accession Primer sequences

number symbol number

______________________________________________________________________

1 CCL2 NM_002982 CCGAGAGGCTGAGACTAAC (s)

TGAAGGTGGCTGCTATGAG (as)

2 CCL3 NM_002983 GACACGGGCAGCAGACAG (s)

GCAGAGGAGGACAGCAAGG (as)

3 CCL5 NM_002985 CTGTCATCCTCATTGCTACTG (s)

CACTTGGCGGTTCTTTCG (as)

4 CXCL10 NM_001565 AAGAAGGGTGAGAAGAGATGTC (s)

TAGGGAAGTGATGGGAGAGG (as)

5 FGF2 NM_002006 TGCTGGTGATGGGAGTTG (s)

GGTCCGTCCTGAGTATTCG (as)

1. GAPDH NM_002046 TGGTCTCCTCTGACTTCAAC (s)

GTAGCCAAATTCGTTGTCATAC (as)

1. GCSF NM_000759 TGGCAGCAGATGGAAGAAC (s)

GGAAAGCAGAGGCGAAGG (as)

8 GMCSF NM_000758 TCCTGAACCTGAGTAGAGAC (s)

CAGTGCTGCTTGTAGTGG (as)

9 IFNG NM_000619 GGTTCTCTTGGCTGTTACTG (s)

TCTGTCACTCTCCTCTTTCC (as)

10 IL-1b NM_000576 TGGCTTATTACAGTGGCAATG (s)

GTGGTGGTCGGAGATTCG (as)

11 IL-6 NM_000600 TGAGAGTAGTGAGGAACAAG (s)

CGCAGAATGAGATGAGTTG (as)

12 IL-13 NM_002188 ATCACCCAGAACCAGAAGG (s)

AGAATCCGCTCAGCATCC (as)

13 IL-16 NM_001172128 CAACTCCTCCACTGACTCTG (s)

CCTGCCGACATCTTCTCC (as)

14 IL-17 NM_002190 ACTGCTACTGCTGCTGAG (s)

GAGATTCCAAGGTGAGGTG (as)

15 LIF NM_002309 CCAACAGCAAGACGAGGATG (s)

GATGAAGCAGGAAGGAGAAGG (as)

16 PDGFB NM_002608 CACCAGTCACCTCGTCTC (s)

TCTACAGCCACCTCAACAG (as)

17. TNF NM_000594 TCAGCAAGGACAGCAGAG (s)

GTATGTGAGAGGAAGAGAACC (as)

18. VEGF NM_001025366 GGCGAAGAGAAGAGACACATTG (s)

GGAGCAGGAAGAGGATGAGG (as)

_________________________________________________________________________

as, anti-sense. s, sense.
