# Supplementary material for: Profiles of cytokines secreted by isolated human endometrial cells under the influence of chorionic gonadotropin during the window of embryo implantation
Source: Reprod Biol Endocrinol. 2013 Dec 17;11:116. doi: 10.1186/1477-7827-11-116 (PMC3878507; doi:10.1186/1477-7827-11-116)
Supplement: Additional file 4: Table S4 — Profiles of secreted cytokines, chemokines and growth factors from isolated endometrial cells of three culture groups in the basal condition. [file 1477-7827-11-116-S4.doc]

**Additional file 4: Table S4 Profiles of secreted cytokines, chemokines and growth factors from isolated endometrial cells of three culture groups in the basal condition**^1^

________________________

Name of Groups

cytokine 1 2 3

_________________________

CCL2 0 3 3

CCL3 3 0 2

CCL4 4 3 3

CCL5 3 4 3

CCL7 3 0 0

CCL11 1 2 1

CCL27 0 0 0

CXCL1 4 2 2

CXCL9 3 3 3

CXCL10 5 2 2

CXCL12 3 3 3

FGF2 3 3 3

GCSF 0 0 4

GMCSF 4 4 3

HGF 3 3 3

IFNa2 2 2 2

IFNG 3 2 2

IL-1a 2 2 2

IL-1b 2 1 1

IL-1ra 3 2 2

IL-2 0 0 1

IL-2ra 3 3 3

IL-3 2 2 2

IL-4 0 0 1

IL-5 0 0 0

IL-6 5 3 5

IL-7 0 0 2

IL-8 0 4 4

IL-9 0 0 3

IL-10 0 0 0

IL-12p40 3 3 3

IL-12p70 3 0 0

IL-13 1 0 1

IL-15 0 0 0

IL-16 2 2 2

IL-17 2 1 0

IL-18 0 0 0

LIF 3 2 2

LTA 0 0 0

MCSF 3 3 3

MIF 4 4 4

bNGF 0 0 0

PDGFbb 3 0 0

SCF 0 0 0

SCGF 4 0 0

TNF  1 0 2

TRAIL 2 2 2

VEGF 4 3 4

___________________________________________________________________________

Group 1, epithelial cells. Group 2, stromal cells. Group 3, mixed cells. Scales for profiles (pg per μg total protein): 0, not detectable; 1, >0.05 - 0.1; 2, >0.1 – 1.0; 3, >1.0 – 10.0; 4, >10.0 – 100; 5, >100.0. ^1^cells grown to ~80% confluency and maintained in serum-free, DMEM-F12 (1:1) medium supplemented with insulin (10 μg/ml), transferrin (5.5 μg/ml), selenium (6.7 ng/ml), hydrocortisone (5 μg/ml) and gentamicin (10 μg/ml), penicillin (100 IU/ml), streptomycin (100 μg/ml) and fungizone (2 μg/ml).
